# The African context of the Cape Town Declaration

**Published:** 2018

**Authors:** Zilla Peter, Zühlke Liesl, Sliwa Karen, Commerford Patrick

**Affiliations:** Chris Barnard Division of Cardiothoracic Surgery, Groote Schuur and Red Cross Children’s Hospital, University of Cape Town, Cape Town, South Africa; Division of Paediatric Cardiology, Department of Paediatrics, Red Cross Children’s Hospital; Division of Cardiology, Department of Medicine, Groote Schuur Hospital, University of Cape Town, Cape Town, South Africa; Hatter Institute for Cardiovascular Research in Africa, Department of Cardiology and Internal Medicine, University of Cape Town; Division of Cardiology, Department of Medicine, University of Cape Town and Groote Schuur Hospital, Cape Town, South Africa; Editor-in-Chief (CVJA)

This issue of the *Cardiovascular Journal of Africa* publishes the Cape Town Declaration (page 256) simultaneously with eight other journals worldwide.^1^ It is a call to arms within the cardiac surgical community.

A decade after the late Professor Bongani Mayosi led the Pan-African Cardiology of Africa (PASCAR) call and alerted the world to the huge public health crisis of rheumatic heart disease (RHD) in the Drakensberg Declaration,^2^ the cardiac surgery community has also recognised the magnitude of the problem, aggravated by the lack of cardiac surgery in the most affected regions of the world. When representatives of all major cardiothoracic societies, industry, civic organisations as well as surgeons from all over the world convened to celebrate the 50th anniversary of the first heart transplant in Cape Town, an entire day was dedicated to a south–north dialogue, focusing on the unmet needs of cardiac surgery in low-income countries.

Nowhere is this unmet need more glaring than on the African continent. In most countries, as far as children are concerned, this lack of life-saving therapy in the absence of a curative alternative has been only sporadically relieved by fly-in missions from high-income countries, bringing teams of specialists and consumables to remote places to operate on a handful of children, before departing. For RHD, however, it is adolescents and young adults who represent the majority of patients in need of cardiac surgery among the more than six billion people living outside high-income countries. Although their numbers approximate those of patients with HIV^3^ (World Health Organisation Global Health Observatory data 2017), to date, this startling comparison has been largely ignored globally. There has been little RHD activism similar to the one that drove the worldwide HIV campaigns, until recently.

Of all the efforts to create awareness for the neglected millions of patients with RHD, the tireless work of Bongani Mayosi stands out.^1^-^4^,^6^-^9^ His focus on cardiovascular diseases of the poor in Africa led to South Africa becoming an epicentre of research on RHD, with other internationally renowned scientists following in his footsteps.^1^,^3^-^5^,^7^-^9^ Therefore, it seems fitting that all the international cardiac surgical societies came to Cape Town to not only celebrate a medical event that happened here 50 years ago and that was unparalleled in its impact, but to finally unite the cardiac surgical community behind the plight of those living with and affected by RHD. Furthermore, in May 2018, the World Health Assembly resolution against RHD was finally adopted, following the African Union communique spearheaded by Mayosi in 2016.^7^ We stand at a powerful new juncture in the fight against rheumatic heart disease, led by those living in RHD-endemic countries.

As a first concrete result of this gathering of leaders from all over the world, which led to the Cape Town Declaration, an analysis of the cardiac surgical capacity of 16 different countries will soon be published.^8^ More importantly, this analysis for the first time provides a needs assessment in affected regions. Although echocardiographic screening studies had provided better insight into the burden of asymptomatic RHD, hardly any data existed on the proportion of patients actually needing cardiac surgery. Furthermore, cohort studies from tertiary institutions had already started to highlight one inconvenient truth for health politicians: although the incidence of rheumatic fever has significantly dropped, the drastic cuts to financing cardiac surgery in the public domain of countries such as South Africa are not justified, since both the incidence of RHD among adults and the need for surgery remain high.^9^ Yet, while the indigent population of South Africa and the Maghreb has at least some limited access to heart valve surgery, the majority of Africans south of the Sahara has none.

What makes the plight of Africa so particularly sobering in this regard is that the cardiac surgery dilemma is not only a reflection of economic prowess. In our comparison of 16 different countries being home to four billion people, there was only a weak correlation between the per capita GDP and the provision of cardiac surgery. With a near-identical per capita GDP, Iran and South Africa spend nearly identical per capita amounts on health, yet Iran performs 525 cardiac operations per million, as opposed to 142 in South Africa. Likewise, Nigeria’s per capita GDP is almost a third higher than that of India, but India delivers 154 cardiac operations per million, as opposed to 0.5 in Nigeria.^8^

Similarly, the Gini index, as an expression of societal inequality, does not always correlate with access to cardiac surgery: Algeria, with a Gini index of 28 and South Africa with 63, provide similar levels of cardiac surgery (127/million versus 142/million operations). Yet, an access ratio between affluent private patients and indigent patients of 12 in South Africa but only 1.2 in Algeria does correlate with the extremely divergent Gini indices between these two countries.^8^ In that regard, South Africa is at the extreme end: those 17% of the population who have access to private medical care receive 595 cardiac operations per million, while the 83% of the population who depend on public medical care receive only 50 operations per million per year.

Alternatively expressed, one private hospital cares for 300 000 cardiac surgical patients who have medical insurance (with 7.1 cardiac surgeons/million), as opposed to 6.1 million patients per centre who depend on the public sector (with 0.7 cardiac surgeons/ million).^8^ In an even more blunt comparison, South Africa’s almost 50 private cardiac centres serving a population of 10 million medical aid patients are in stark contrast to an indigent population of a billion people living in sub-Saharan Africa, with access to half this number of hospitals offering heart valve surgery.

Changing this appalling state of affairs will take huge efforts on many levels. Pressure on governments will need to be coordinated to have any effect. Activist groups such as RHD Action will need to be broadly supported. International awareness needs to increase dramatically and the readiness of the medical device industry to become a partner and adjust their price policy to indigent patients and not only to the African private sector will be paramount. Advice from health economists needs to be sought to provide cost-effective, evidence-based interventions and present a high-level business model to international agencies such as the World Heart Federation and World Health Organisation.

Once these prerequisites are in place, training specialists in a country that has exposure to RHD, rather than in North America or Europe, will be crucial, with the goal firmly focused on local capacity building. Critically important will be political will and funding to drive a unified and integrated RHD agenda. These key demands have been formulated in the Cape Town Declaration. Much depends now on the support it gets to implement them. One thing is undisputed: the time to act is now!
